# Two-Step Epimerization of Deoxynivalenol by Quinone-Dependent Dehydrogenase and *Candida parapsilosis* ACCC 20221

**DOI:** 10.3390/toxins15040286

**Published:** 2023-04-16

**Authors:** Yuqian Tang, Dingna Xiao, Chendi Liu

**Affiliations:** School of Food Science and Engineering, South China University of Technology, Wu Shan, Guangzhou 510640, China

**Keywords:** deoxynivalenol, epimerization, QDDH, *Candida parapsilosis* ACCC 20221

## Abstract

Deoxynivalenol (DON), one of the main mycotoxins with enteric toxicity, genetic toxicity, and immunotoxicity, and is widely found in corn, barley, wheat, and rye. In order to achieve effective detoxification of DON, the least toxic 3-epi-DON (1/357th of the toxicity of DON) was chosen as the target for degradation. Quinone-dependent dehydrogenase (QDDH) reported from *Devosia train* D6-9 detoxifies DON by converting C3-OH to a ketone group with toxicity of less than 1/10 that of DON. In this study, the recombinant plasmid pPIC9K-QDDH was constructed and successfully expressed in *Pichia pastoris* GS115. Within 12 h, recombinant QDDH converted 78.46% of the 20 μg/mL DON to 3-keto-DON. *Candida parapsilosis* ACCC 20221 was screened for its activity in reducing 86.59% of 3-keto-DON within 48 h; its main products were identified as 3-epi-DON and DON. In addition, a two-step method was performed for epimerizing DON: 12 h catalysis by recombinant QDDH and 6 h transformation of the *C. parapsilosis* ACCC 20221 cell catalyst. The production rates of 3-keto-DON and 3-epi-DON were 51.59% and 32.57%, respectively, after manipulation. Through this study, effective detoxification of 84.16% of DON was achieved, with the products being mainly 3-keto-DON and 3-epi-DON.

## 1. Introduction

Deoxynivalenol (DON), also known as vomitoxin, is a toxic metabolite produced by *Fusarium* sp. that has appeared in grains, food products, and feeds worldwide in recent years. A total of 821,100 samples collected from 74 countries were analyzed over a 10-year period, and DON was detected in 64% of all samples. DON detection was most prevalent in samples from East Asia, at 84.8% [1]. The United States Food & Drug Administration (US-FDA) has set a limit of 1000 μg/kg for wheat products (e.g., bran, flour, and germ) for human consumption. A 15-year study examined 76 samples from four crop-producing regions in Brazil and found DON contamination levels as high as 94%, with levels ranging from 1700 to 7500 µg/kg [2]. Among 181 wheat samples from northwest China, the DON contamination rate was 82.9%, with an average concentration of 500 μg/kg, of which 10% exceeded the Chinese standard of 1000 μg/kg [3]. DON has enteric toxicity, genetic toxicity, and immunotoxicity [4], and has become the primary measure of food safety. It is difficult to reduce its toxicity during food processing and storage because of its stable chemical properties and high heat resistance. It can be enriched in the food chain for a long time and can seriously threaten the health of people and animals. Effective control and elimination of DON in cereals and their products are very important for food production and food safety.

Although many physical and chemical methods have been developed to reduce or eliminate DON in grains, few technologies meet the requirements of practical application owing to their limitations. Physical methods are difficult and less efficient in handling large batches of products. Chemical methods may reduce the nutritional quality and organoleptic properties of food products, and there is some environmental pollution. Biological detoxification methods are favored because of their advantages of high efficiency, high specificity, and environmental protection.

The study of biodegradation of DON started with the discovery of mixed microbial groups. Guan et al. [5] obtained anaerobic microbial mixtures from animal intestines with a degradation rate of >90% for DON. Wang et al. [6] isolated a bacterial colony IFSN-C1 from wheat leaves that can transform DON into 3-keto-DON and 3-epi-DON. The separated mixed strains, which seem to have good degradation efficiency, can only work under strict anaerobic conditions that are often limited in actual production. Most mixed bacteria include several strains that have not been confirmed to be safe and that have a clear mechanism of effective cooperation in DON detoxification.

At present, domestic and overseas studies on DON biodetoxification have mainly focused on screening single microorganisms with detoxification ability from the environment, such as *Bacillus* sp. [7], *Devosia* sp. [8,9,10,11], *Nocardioides* sp. [12], *Paradevosia* sp. [13], *Sphingomonas* sp. [14], *Pelagibacterium* sp. [15], *Aspergillus tubingensis* [16,17], and *Aspergillus oryzae* [18]. Among them, researchers have reported that many *Bacillus* (*Bacillus cereus* [19], *Bacillus subtilis* [20,21], and *Bacillus circulans* [7]) can degrade 71.40–98.85% of DON in feed within 72 h without metabolites and toxicity. The only one whose degradation pathway, metabolites, and key enzymes have been identified is *Devosia* sp., which achieves a >88% degradation rate of 20–500 μg/mL DON in 2–72 h [8,9,10,11,22,23,24,25]. In addition to bacteria, *Aspergillus tubingensis* [16,17] and *Aspergillus oryzae* [18], as other fungi, have also been reported to decompose >90% of 1–50 μg/mL DON in 14 days.

Screening for catalytically competent environmental microorganisms is highly uncertain, and conventional enrichment of the initial screen will silence the functional gene for catalytic DON under laboratory culture conditions. For example, the mycotoxin degradation performance of isolated strains is unstable, which makes previous efforts ineffective for screening or studying. Yu et al. [26] screened two batches of mixed cultures from chicken intestines with a de-epoxy effect on DON, which had a high transformation effect during the first two generations of culture. However, the degradation effect fell precipitously in the third generation, and was almost 0% after five generations, while two isolates lost degradation activity in culture after 3–4 passages.

In addition to the low degradation efficiency of a single strain, the heavy screening workload results in a long duration needed for digging key enzymes, controlling metabolite formation, and so on. Furthermore, the safety of the strains is not guaranteed before screening, which greatly adds uncertainty to the results. To date, only one bacterial strain, *Eubacterium* sp. BBSH797, has been approved as a feed additive, but the enzyme has not been used to detoxify DON in foods [27].

**Figure 1:**
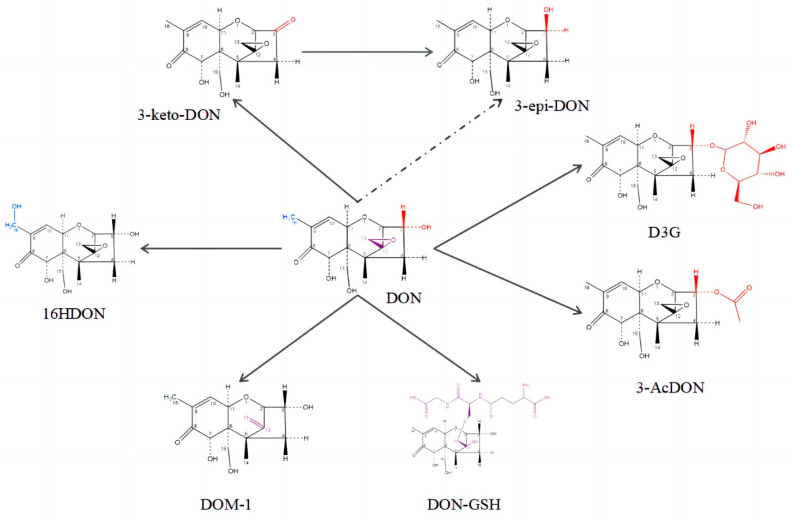
The targets for degradation of DON and the structures of metabolites.

The structure of DON is relatively complex, with the C12-13 epoxy group and hydroxyl groups C3, C7, and C15 the main toxic groups [28]. Among the reported DON derivatives through various transformation pathways [29,30] (Figure 1), the order of toxicity is 15Ac DON > DON > 3-keto-DON ≥ 3Ac DON > DOM-1 > 3-epi DON based on the results of animal cell experiments [31]. The decyclation of the C12- and 13-position epoxy rings and isomerization of the C3-position hydroxyl groups of DON play an important role in its toxicity reduction [32,33,34]. Compared with DON, 3-epi DON, DOM-1, and 3-keto-DON have significantly reduced toxicity and have been verified to not release DON or other toxic derivatives in the intestine (i.e., mycotoxins, such as DON-3-Glc and 15-A-DON) [35,36]. However, there are very few reports regarding microorganisms that can reduce DON to low-toxicity DOM-1 and 3-epi-DON.

Therefore, this study attempted to achieve enzymatic transformation from DON to less toxic metabolites. Based on the research results of He [8,10,24] and Carere et al. [9,37], taking the gradual degradation mode of DON in *Devosia* sp. and *Sphingosaminomide* genus as a reference, the C3-OH of DON was first oxidized to generate 3-keto-DON and then further epimerized to 3-epi-DON to detoxify this mycotoxin. Among the reported wild bacteria of *Devosia* sp., both D6-9 [8] and 17-2-E-8 [10] were found to convert DON to 3-keto-DON and further catalyze the isomeric reduction of 3-keto-DON to 3-epi-DON. Carere et al. [9,37] and He et al. [8,38] cloned from *Devosia* sp. the enzymes involved in the C3-OH oxidation and isomerization reduction reactions. The two enzymes that could achieve the first step of conversion, QDDH and DepA, had more than 99% protein sequence similarity and belonged to PQQ-dependent alcohol dehydrogenases, so one of them was selected for the follow-up study in this experiment. For *Sphingosaminomide*. S3-4 was also reported to have the ability to isomerize DON, but He et al. [14] only cloned the enzyme gene AKR18A1 from S3-4 that completes the first step of oxidation reaction, which belongs to aldo–keto reductase.

To oxidize the C3-OH of DON, AKR18A1 and QDDH were selected for exogenous expression, and recombinant QDDH could convert 70% DON to 3-keto-DON after 12 h reaction at 37 °C, which was more efficient than AKR18A1. The second stage was fulfilled by *Candida parapsilosis* ACCC 20221, selected from several *Candida species*, and 3-keto-DON was further isomerized and reduced to form a less toxic 3-epi-DON. As a two-step reaction was carried out by combining recombinant hydroxy oxidase and ACCC 20221, 86.59% DON (20 μg/mL) was converted within 48 h, and the yields of 3-keto-DON and 3-epi-DON were 51.59% and 32.57%, respectively. Finally, we achieved effective detoxification of 84.16% of DON by a two-step process using bioenzymes and yeast, the products of which were mainly 3-keto-DON and 3-epi-DON.

## 2. Results

### 2.1. Recombinant Oxidase Expressed in Pichia pastoris

After adjusting the gene according to the codon preference of yeast genus, the codon adaptation index (CAI) of AKR18A1 increased from 0.43 to 0.95; that of QDDH increased from 0.58 to 0.94, which was conducive to improving its expression level in *Pichia pastoris*. The codon of 78.2% AKR18A1 was optimized, and this portion of QDDH accounted for 70.1%. Among them, the optimized codons mainly focused on coding for amino acids alanine, glycine, isoleucine, leucine, proline, arginine, serine, threonine, valine, and tryptophan, mainly because the codons encoding these amino acids in *Pichia pastoris* were more inclined to GCT, GGT, ATT, TTG, CCA, AGA, TCT, ACT, GTT, and TGA [39,40,41]. The high-frequency codon substitution facilitated the expression of exogenous target proteins in the host *Pichia pastoris*.

**Figure 2:**
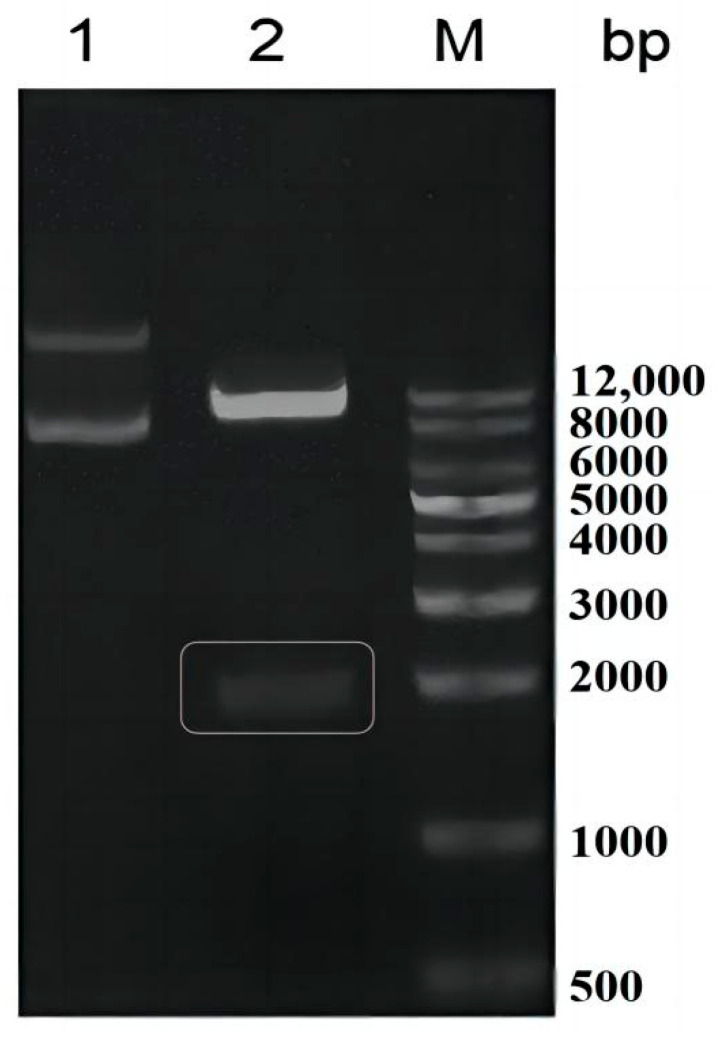
Identification of pPIC9K-QDDH by double digestion: 1 and 2 are before and after pPIC9K-QDDH double digestion; M, DL10000 DNA Marker.

As shown in Figure 2, lane 1 is the recombinant plasmid pPIC9K-QDDH before enzyme digestion with EcoR I and Not I, which have multiple bands due to their annular and superhelical conformations. As shown in lane 2, pPIC9K (9276 bp) and QDDH (1797 bp) bands were obtained after double-enzyme digestion. Compared with the molecular weight protein marker, the bands in lane 2 were in the expected range of 8000–10,000 bp and 1000–2000 bp. Subsequent sequencing results showed that the optimized QDDH gene was inserted exactly to construct the recombinant plasmid pPIC9K-QDDH. However, subsequent expression experiments showed that recombinant AKR18A1 did not catalyze the oxidation of DON to produce 3-keto-DON.

The linearized plasmid pPIC9K-QDDH was transferred into GS115, and after MD (Minimal Dextrose) agar plates screening and colony PCR identification, the positive recombinant yeast colony with high expression level of the target gene was selected and cultured for 36 h in a shaker flask before 48 h methanol induction.

**Figure 3:**
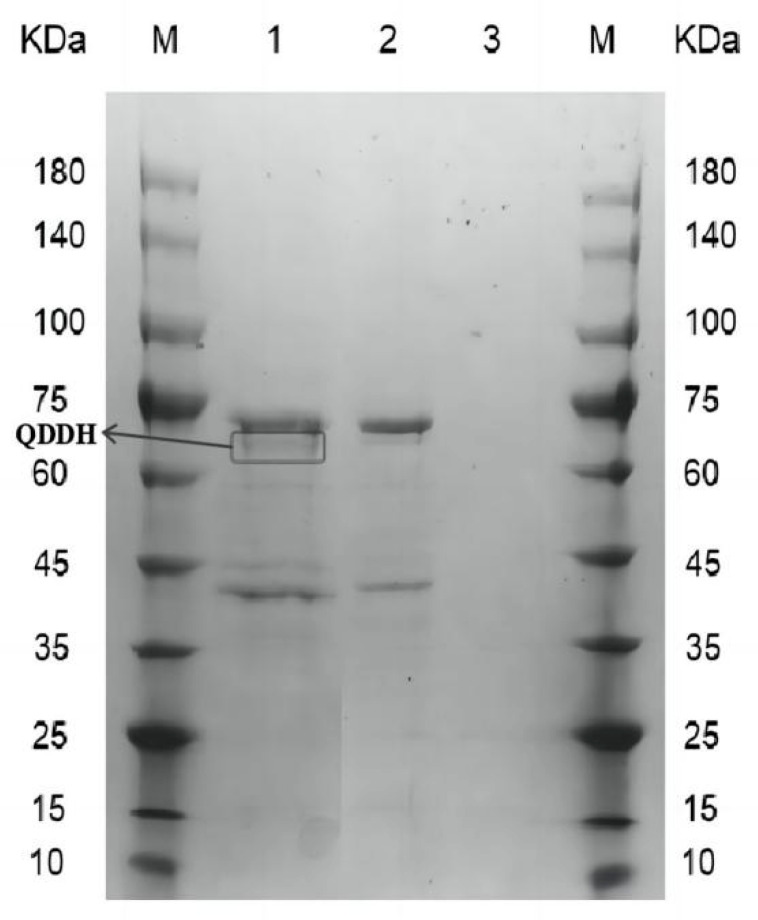
SDS–PAGE analysis of recombinant enzyme: 1, GS115/pPIC9K-QDDH; 2, GS115/pPIC9K; 3, GS115; M, 10 kDa–180 kDa protein marker.

The supernatant was precipitated and analyzed using 12% SDS–PAGE. As shown in Figure 3, the supernatants of GS115/pPIC9K and GS115 were used as controls, and sample 1 was the supernatant of GS115/pPIC9K-QDDH. A protein band, which was significantly different to that of the control, was found in sample 1 in the range of 60–75 kDa. The protein band was consistent with the theoretical size of QDDH (64 kDa), confirming the successful expression of oxidase QDDH in *Pichia pastoris* and its secretion into the extracellular supernatant.

### 2.2. Oxidation of DON to 3-keto-DON

In order to produce the diastereomeric 3-epi-DON by stereochemically inverting the C3 hydroxyl group from the (S) conformation to the (R) conformation, detoxifying the DON-like *Devosia mutans* 17-2-E-8, we used a recombinantly expressed oxidase to complete the first step of the oxidation reaction.

**Figure 4:**
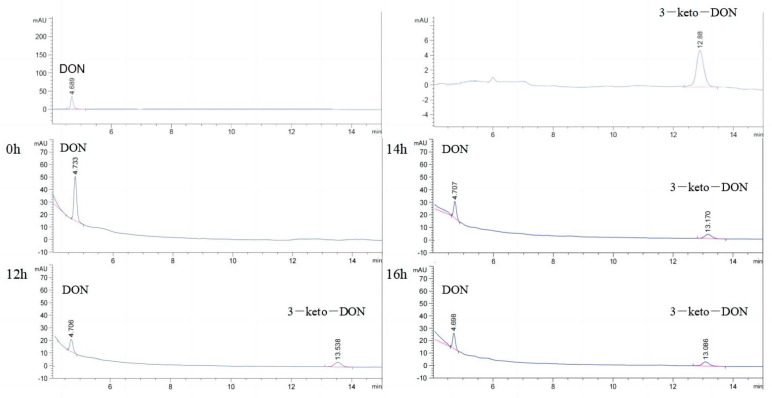
HPLC chromatograms of QDDH reaction system at different keto time points.

**Figure 5:**
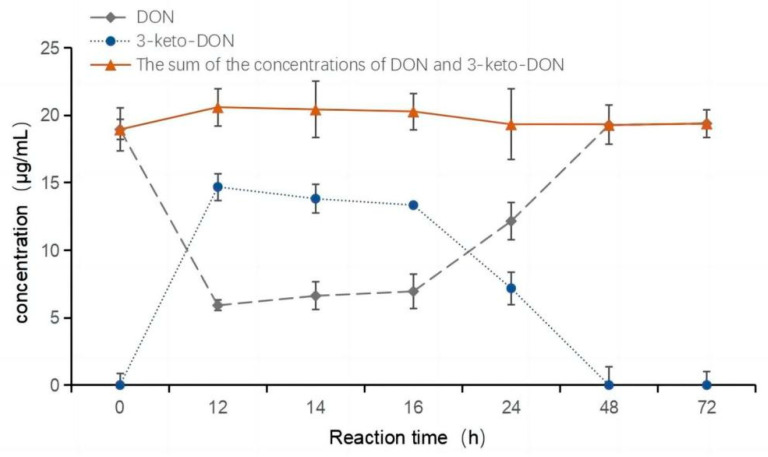
Changes in DON and 3-keto-DON concentrations.

As shown in Figure 4, DON (20 μg/mL) was coincubated with QDDH enzyme solution (460 μg/mL, PQQ) for 72 h. The peak height of DON decreased at 12 h, and then increased with increasing reaction time. The peak area of each substance was compared to a standard curve for statistical comparison and plotting. As shown in Figure 5, the DON concentration decreased significantly after the start of the reaction, from 20 to 5.91 μg/mL during the first 12 h. The corresponding 3-keto-DON concentration increased from 0 to 14.69 μg/mL. The total concentration of the two was maintained at 20 μg/mL. After 12 h, the DON concentration stopped declining and began to increase slowly. The concentration of 3-keto-DON slightly decreased. At the 16 h time point, a notable reduction in the concentration of 3-keto-DON was observed, concomitant with an increase in the concentration of DON. The maximum conversion rate of DON was achieved at the 12 h time point, with a 70% conversion rate and a concentration of 14.69 μg/mL for 3-keto-DON. Over the course of the reaction, the total concentrations of DON and 3-keto-DON remained similar to the initial concentration of DON, and no other products were detected. However, with extension of the reaction time, the accumulated transformation product 3-keto-DON was converted back to DON, weakening the catalytic effect of oxidase QDDH on the generation of 3-keto-DON. To achieve the highest DON biotransformation rate, the reaction was terminated immediately after 12 h.

The enzyme QDDH we used was a PQQ-dependent alcohol dehydrogenase that oxidizes the C3 OH group of DON to form 3-keto-DON, which not only oxidizes alcohols to aldehydes, but also catalyzes the reversible reduction of ketones or aldehydes to their corresponding alcohols [42,43], such as retinol and retinal [44]. In addition, most of the same type of dehydrogenases are reversible, similar to amino acid dehydrogenase (AADH), which catalyzes the reversible reduction of ketoacids and ammonia to produce chiral amino acids [45].

### 2.3. Highly Active Candida parapsilosis Strain Catabolizes 3-keto-DON into 3-epi-DON

This process, known as “DON epimerisation” (Dep), takes place in two stages. The second enzyme reduces 3-keto-DON at the re-face of this stereogenic center to produce 3-epi-DON, which requires the reduction and isomerization of the 3-keto group to generate a hydroxyl group by aldo–keto reductase. As shown in Table 1, aldo–keto reductases have a wide range of microbial sources. Yeast is the most studied microorganism for biological catalysis of ketone reduction, such as *Candida parapsilosis* [46,47,48,49,50,51], *Yarrowia lipolytica* [52,53,54], *Pichia pastoris* [55,56], and *Candida magnolia* [57,58]. The aldo–keto reductase found in *Candida parapsilosis* CCTCC M203011 [46,47] and *Yarrowia lipolytica* CICC 32187 [53] can restore 2-Hydroxyacetophenone as (s)-phenyl ethanediol to provide important chiral intermediate for pesticide, medicine, and electronic materials.

Fungi, such as *Geotrichum candidum* [59], have also been reported to produce carbonyl reductase, whereas bacteria include *Rhodococcus* [60,61,62,63], *Acetobacter* [64], and *Lactobacillus kefir* [65]. We tested the carbonyl reductase activity of a variety of yeasts and bacteria. Five yeast strains were selected experimentally, including *Saccharomycopsis lipolytica* GDMCC 2.197, *Yarrowia lipolytica* GDMCC 2.187, *Candida parapsilosis* GDMCC 2.190, *Pichia pastoris* GS115, and *Candida parapsilosis* ACCC 20221.

**Table 1:** Aldo–keto reductase from microorganisms.

Strains	Enzyme	Substrates	Main Products	References
*Candida parapsilosis*	SCRII	2-hydroxyacetophenone	(S)-phenyl ethanediol	[46,47]
	CpCR	Benzaldehyde	(R)-Benzylalcohol	[49]
	CPR-C1	ethyl ketopantoyl lactone	ethyl d-pantoyl lactone	[51]
	CPR-C2	ketopantoyl lactone	d-pantoyl lactone	[48]
*Yarrowia lipolytica*	YaCRI	2-hydroxyacetophenone	(S)-1-phenyl-1,2-ethanediol	[53]
	YaCRⅡ			
	YlCR	2-propanol	(R)-2-chloro-1-phenylethol	[54]
	YlCR2	COBE *	(S)-CHBE *	[52]
*Candida magnoliae*	CmCR	COBE *	(S)-CHBE *	[57]
	S1	COBE *	(S)-CHBE *	[58]
*Pichia pastoris*	PsCRI	COBE *	(S)-CHBE *	[56]
	PsCRII	COBE *	(S)-CHBE *	[55]
*Rhodococcus R6*	ReADH	2-hydroxyacetophenone	(R)-1-phenyl-1,2-ethanediol	[60]
*Rhodococcus pyridinivorans*	-	para-acetylphenol	S-1-(para-hydroxyphenyl)ethanol	[61]
	RpCR	COBE *	(S)-CHBE *	[62]
*Rhodococcus* sp. *ECU1014*	RhCR	epsilon-ketoester ethyl 8-chloro-6-oxooctanoate	ethyl (S)-8-chloro-6-hydroxyoctanoate	[63]

* notes: COBE: 4-chloro-chloroacetoacetate; (S)-CHBE: (S)-4-chloro-Chloro-3-Hydroxybutanoate; (R)-CHBE: (R)-4-chloro-chloro-3-hydroxybutyrate.

Whole–cell catalysts were added to phosphate buffer containing 20 μg/mL 3-keto-DON standard. As shown in Table 2, the 3-keto-DON conversion rates of *Y. lipolytica* GDMCC 2.187, *C. parapsilosis* ACCC 20221, and *P. pastoris* GS115 were 100%, 86.59%, and 58.60%, respectively. They converted 3-keto-DON into two main products; one had the same retention time as the DON standard sample, and the other was speculated to be 3-epi-DON, because its retention time was earlier than that of DON according to He et al. [8]. The maximum absorption wavelength of the early retention-time product was consistent with that of 3-epi-DON.

The conversion rates of 3-keto-DON in *S. lipolytica* (GDMCC 2.197) and *C. Parapsilosis* (GDMCC 2.190) were 37.13% and 30.21%, respectively. However, they were transformed into only one product and judged to be DON, which was probably the result of S-carbonyl reductase in the strains. Despite the reportedly high carbonyl reductase activity of *Rhodococcus*, *Rhodococcus opacus* PD 630 had no effect on 3-keto-DON.

As shown in Table 2, *C. Parapsilosis* ACCC 20221 was able to convert 86.59% of 3-keto-DON, and the proportion of 3-epi-DON in the product was higher than that for *Y. Lipolytica* GDMCC 2.187 and *P. pastoris* GS115; therefore, for subsequent experiments, *C. Parapsilosis* ACCC 20221 was used to study epimerization in DON.

### 2.4. Catabolite Identification of 3-keto-DON by LC–MS

**Figure 6:**
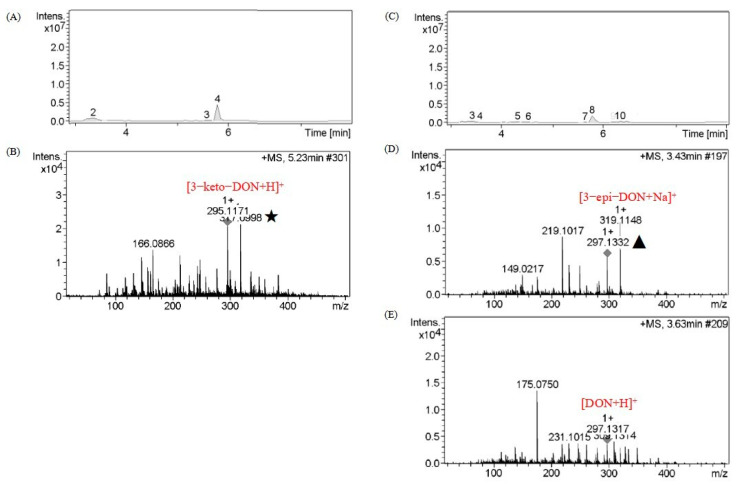
Liquid chromatography–mass spectrometry of 0 h and 48 h reaction liquid for the degradation of 3-keto-DON. (**A**) 0 h; (**B**) Component 4 of 0 h; (**C**) 48 h; (**D**) Component 3 of 48 h; (**E**) Component 4 of 48 h.

As shown in Figure 6, 3-keto-DON (molecular weight of 294.3) corresponded to the fourth component of the total ion flow, and the mass-to-charge ratios of the two peaks in positive ion mode were 295.1171 and 317.0998 (★), corresponding to [3-keto-DON+H]^+^ and [3-keto-DON+Na]^+^.

After the 48 h reaction, the total ion flow in Figure 6 appeared at approximately 3.5 min; the transformation products included the third and fourth components. The mass–charge ratios of the two peaks in the third component were 297.1332 (▲) and 319.1148, corresponding to [M+H]^+^ and [M+Na]^+^, respectively. Therefore, the molecular weight of the third component was approximately 296. The highest mass–charge ratio of the fourth component was 297.1317, corresponding to [M+H]^+^; the molecular weight of the product was approximately 296. Components 3 and 4 had the same molecular weight but different retention times.

According to Hassan et al. [23,66], DON and 3-epi-DON are isomers of each other, with a molecular weight of 296.3, but owing to differences in the conformation of their C3 hydroxyl groups, the polarity of 3-epi-DON is greater than that of DON, and its retention time is earlier than that of DON in the C18 reverse column [12,32,66,67]. Based on LC–MS, it was inferred that components 3 and 4 were 3-epi-DON and DON, respectively.

The 3-keto-DON was transformed into 3-epi-DON and DON simultaneously, which were catalyzed by aldehyde-ketone reductases from yeast, as their reduction reactions can form two alcohol configurations (S type, R type). Usually, these two types of enzymes are expressed simultaneously in one strain [68]. For example, *Candida parapsilosis* CCTCC M20301 [46,47] contained both (R)- and (S)-aldo–keto reductases (RCR and SCR), which produced both (R)-phenyl glycol and (S)-phenyl glycol when acted on substrates 2-hydroxyacetophenone. The flow direction of the product depends on the relative activities of the two enzymes and the utilization of coenzymes.

Therefore, DON (S-type) and 3-epi-DON (R-type) [66] were generated simultaneously in this yeast whole-cell catalytic reduction of 3-keto-DON, which requires further microbial metabolic regulation of the biological synthesis preference for R-type aldehyde-ketone reductase to convert 3-keto-DON to 3-epi-DON in large diastereomeric ratios. Subsequently, we plan to discover a new efficient R-aldehyde-ketone from *C. Parapsilosis* ACCC 20221, or heterologous expression of R-aldehyde-ketone reductase, to increase the efficiency of bioconversion.

### 2.5. Epimerization of DON

Previously, we converted DON to 3-keto-DON by recombinant QDDH with NADPH and completed enzymatic epimerization to 3-epi-DON by *C. parapsilosis* ACCC 20221 whole cells. In this experiment, recombinant QDDH was added at 0 h in a phosphate buffer system containing 20 μg/mL DON and was inactivated after 12 h. *C. parapsilosis* ACCC 20221 whole cells were then added, and the reaction was continued for 36 h.

**Figure 7:**
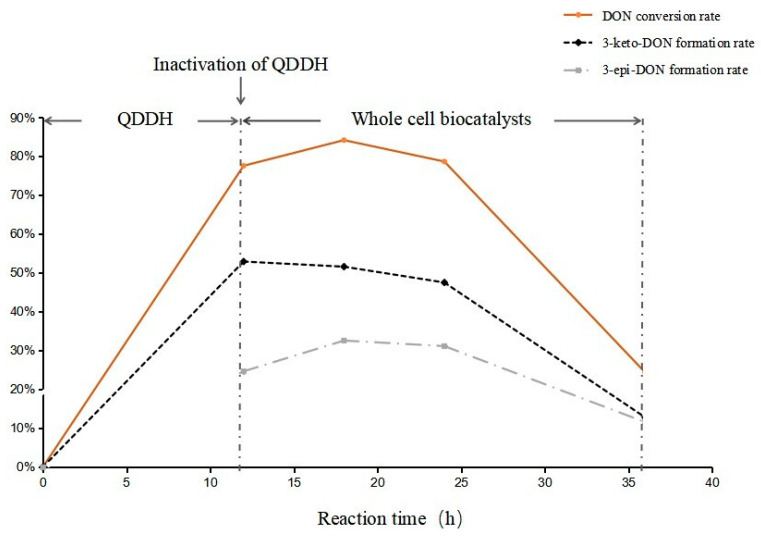
Mutual transformation of DON and its products.

As shown in Figure 7, after oxidase QDDH interacted with DON for 12 h, 77.56% of DON was converted, while the 3-keto-DON generation rate was 51.59%. At 18 h of epimerization, the DON conversion rate reached a peak of 84.16%, decreased to 78.65% after 24 h, and then dropped sharply to 24.38%. The maximum 3-keto-DON was produced at 12 h, but then continued to decrease after the addition of yeast whole-cell catalyst; the liquid phase results showed that 3-keto-DON was catalyzed to 3-epi-DON and DON. The 3-epi-DON formation rate peaked at 32.57% after 18 h and then decreased slowly.

## 3. Discussion

DON, one of the most serious mycotoxins causing pollution problems, has stable chemical properties and good heat resistance. Therefore, it is difficult to reduce toxicity during food processing, storage, and traditional treatments. Conventional research on DON biodegradation has mainly focused on screening various environmental microorganisms for DON degradability and analyzing detoxification processes before imitation. However, this extremely complex and highly uncertain process required further investigation before utilization. Therefore, this study attempted to achieve enzymatic transformation from DON to less toxic metabolites in order to shorten this process.

The C12,13-epoxy group is a key toxic group because of the toxicity of various conversion products. However, the C12,13-epoxy group structure is unique, and there have been few reports of successful epoxidation–ring opening. Moreover, DON detoxification by epoxidation–ring opening is difficult under aerobic conditions. Epoxide hydrolase [16] and lipase [17] are reported to be capable of epoxidation–ring opening; however, we failed to degrade DON using this approach.

Based on the structure and toxicity of the other transformation products, we referred to the two-step reaction mode of oxidation of the C3-hydroxyl group of DON by *Devosia* 17-D-E-8 [38] and D6-9 [8], and selected the derivative 3-epi-DON as the target of the conversion pathway. The whole epimerization process consists of two separate steps in which the two catalysts are put in separately.

In the first reaction step, the recombinant plasmid pPIC9K-QDDH was constructed, and proteins were successfully expressed in *Pichia pastoris* GS115, with a molecular weight of 64 kDa. QDDH, which treated DON (20 μg/mL) at 37 °C for 12 h, has the ability to transform 70% DON into 3-keto-DON. However, as time lengthens, the reaction begins to proceed in reverse. We also purchased some other dehydrogenases but did not detect degradation activity.

Thus, in our experiments, in the early stage of the reaction, the substrate DON was sufficient, the reaction was carried out in the direction of generating aldehydes, and after 12 h of reaction, the substrate 3-keto-DON accumulated in large quantities. After 16 h, QDDH exerted its other properties, and the reaction was carried out in the direction of generating alcohols. He et al. [8] reported that *E. Coli* BL21 recombinant expression of QDDH (1.2 g/L) can transform 100 μM DON to form 3-keto-DON in 1 h. However, their reaction times were not prolonged and no reverse conversion of the 3-keto-DON converted by QDDH to DON was observed. Yang et al. [37] recombinantly expressed and purified DepA, which has 99.83% sequence similarities with QDDH; 1.6 μM DepA was able to completely convert 500 μM DON to 3-keto-DON within 12 h. Qin et al. [69] recombinantly expressed DON dehydrogenase (DDH) from *Pelagibacterium halotolerans* ANSP101; 47.0 μg/mL of DDH converted 90.5% of 50 μg/mL DON after 12 h. The reaction did not continue to determine whether 3-keto-DON would be reconverted back to DON in the presence of DepA. In contrast, reactions catalyzed by dehydrogenases are theoretically reversible, and this has been demonstrated in several studies; for example, amino acid dehydrogenase (AADH) can catalyze the reversible reduction of keto acids and ammonia to produce chiral amino acids [45]. Therefore, the conversion of DON catalyzed by a dehydrogenase such as QDDH may also be a reversible reaction; the key points regulating the reaction will be investigated in subsequent experiments. Alternatively, the enzyme could be modified at the genetic level so that the reaction proceeds in the direction of producing 3-keto-DON.

In response to Step 2, all reported enzymes for 3-keto-DON reduction to 3-epi-DON are aldo–keto reductases [8,9]. We tested several commercialized aldo–keto reductases to complete the epimerization, but failed. S-type and R-type aldo–keto reductases generally co-exist in microorganisms, but are expressed at different levels. Most of the commercial aldo–keto reductases acquired were derived from microorganisms directly without separating the two types of aldo–keto reductases. At the same time, we wanted to obtain an efficient whole-cell catalyst that could restore 3-keto-DON into 3-epi-DON; therefore, *Candida parapsilosis* ACCC 20221, which can reduce 3-keto-DON to 3-epi-DON, was screened from yeast to catalyze the second step. The conversion rate of 3-keto-DON reached 86.59% within 48 h. However, more than one enzyme may be at work in this process. He et al. [8] also found two enzymes in the same strain that can convert 3-keto-DON: AKR6B1 and AKR13B2. After 6 h of reacting with 3-keto-DON, the products of the former were only 3-epi-DON, but those of the latter were 3-epi-DON and DON. We could target AKR6B1 and analyzed the differences between the two types of enzymes to identify an enzyme that allows the complete conversion of 3-keto-DON to 3-epi-DON in the future.

In summary, QDDH and *C. parapsilosis* ACCC 20221 whole-cell catalysts were added to the DON reaction system in two steps: after 12 h of reaction with QDDH and 6 h of reaction with whole cells, the production rates of 3-keto-DON and 3-epi-DON were 51.59% and 32.57%, respectively, which could achieve effective detoxification of DON. In the first stage, most of the DON was transferred into 3-keto-DON by recombinant QDDH within 12 h, considering the previous result. To stop the reverse action, recombinant QDDH was inactivated, and the second step was carried out immediately by *C. parapsilosis* ACCC 20221 whole-cell accession, which worked for 6 h. It is speculated that most 3-keto-DON generated 3-epi-DON in this 6 h, and some of the DON may have been absorbed by yeast cells so that the DON conversion rate and 3-epi-DON generation rates all reached their highest levels. Subsequently, the reaction was processed mainly by the R-aldo–keto reductases in ACCC 20221, which decreased the DON conversion rate and metabolite formation. Therefore, the process should be terminated 18 h before new progress is made in order to transform DON into 3-epi-DON.

## 4. Conclusions

In this study, a two-step method was developed to accomplish epimerization detoxification of the hydroxyl group at the C-3 position, the main toxic group of DON, starting from the known low-toxicity product 3-epi-DON, with the main products being 3-keto-DON and 3-epi-DON. The first step was the conversion of DON to the less toxic 3-keto-DON within 12 h by recombinant expression of QDDH. The second step was the conversion of 3-keto-DON to the almost non-toxic 3-epi-DON within 6 h, using the screened *Candida parapsilosis* ACCC 20221. The approach developed in this study can efficiently detoxify DON and may provide a reference for the detoxification of DON in feeds.

## 5. Materials and Methods

### 5.1. Chemicals and Strains

The restriction enzymes, T4 DNA ligase, MiniBEST Plasmid Purification Kit, MiniBEST DNA Fragment Purification Kit, MiniBEST Agarose Gel DNA Extraction Kit, and YeastmakerTM Yeast Transformation System 2 were supplied by TaKaRa Bio (Shiga, Japan). The BCA Protein Quantitative Kit was obtained from Sangon (Shanghai, China). DON and 3-keto-DON standards were purchased from TripleBond (Guelph, ON, Canada). All other chemicals used in this study were of analytical grade. *Pichia pastoris* GS115 and pPIC9K were purchased from Invitrogen (Carlsbad, CA, USA). *Saccharomycopsis lipolytica* GDMCC 2.197, *Yarrowia lipolytica* GDMCC 2.187, and *Candida parapsilosis* GDMCC 2.190 were purchased from the Guangdong Microbial Culture Collection Center (GDMCC). *Candida parapsilosis* ACCC 20221 and *Rhodococcus opacus* PD 630 were gifted by Professor Jiguo Yang of the South China Coordinating Innovation Institute and preserved in our laboratory in a −80 °C refrigerator.

### 5.2. Cloning, Expression, and Purification of AKR18A1 and QDDH in Pichia pastoris

The AKR18A1/QDDH gene selected for transformation of DON to 3-keto-DON was codon-optimized and synthesized by Sangon (Shanghai, China). The desired gene fragments were inserted into the plasmid pUCm-T to obtain pUCm-T-AKR18A1/pUCm-T-QDDH.

**Figure 8:**
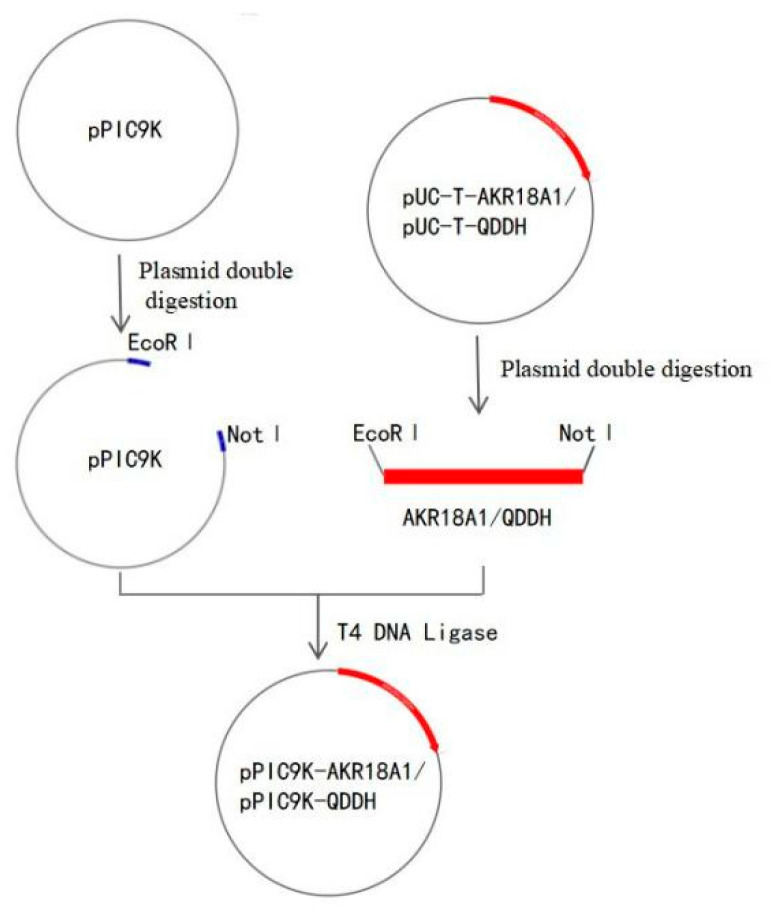
Schematic construction of recombinant plasmids pPIC9K-AKR18A1/pPIC9K-QDDH.

The target plasmid pPIC9K-QDDH was constructed by double-restriction endonucleases, as shown in Figure 8. After simultaneous action of pUC-T-AKR18A1 and pUC-T-QDDH with restriction endonucleases *EcoR I* and *Not I*, the desired gene fragments AKR18A1 and QDDH were purified and ligated with pPIC9K treated with *EcoR I* and *Not I* ligation to construct plasmid pPIC9K-AKR18A1 and pPIC9K-QDDH. The sequencing-validated plasmids were transferred to competent Pichia pastoris GS115 after linearization by Sac I. Positive clone strains were screened by MD (Minimal Dextrose) agar plates and further confirmed by colony PCR. To induce expression of the target protein, 1.5% methanol was added every 24 h during the incubation. After 72 h induction at 28 °C, the fermentation supernatants were collected by centrifugation. Finally, we mixed 20 μL of fermentation supernatants with 5 μL of loading buffer (5×), placed the mixture in a boiling water bath for 5 min, and then added it to 12% SDS–PAGE for detection.

### 5.3. Analysis of Recombinant Protein for DON Oxidation Activity

The purified QDDH protein (400 μL) was assayed for activity in 500 μL of a solution composed of 20 μg/mL DON, 10 μM pyrroloquinoline quinone (PQQ), 1 mM Ca^2+^, and 50 mM phosphate buffer (pH 6.0). The reaction was carried out at 37 °C for 12 h, and terminated by adding an equal volume of methanol. The concentrations of DON and 3-keto-DON were monitored using high-performance liquid chromatography (HPLC).

The DON degradation rate was calculated using the following formula: Percentage decrease in DON = [1 − (remaining DON concentration/initial DON concentration)] × 100.

### 5.4. HPLC Analysis

An Agilent 1260 series HPLC system was used to analyze DON and its metabolized products. An Agilent ZORBAX SB-C18 column (4.6 mm × 250 mm, 5 μm) was used for separation, and the mobile phase consisted of 84% water and 16% acetonitrile at a flow rate of 0.9 mL/min. DON, 3-keto-DON, and 3-epi-DON were detected by monitoring the absorbance at 218 nm; the injection volume was 20 μL for each sample solution and the column temperature was maintained at 30 °C. Concentrations were quantified by comparison with standard substances.

### 5.5. Analysis of Candidate Strains for 3-keto-DON Reduction Activity

Five strains were screened as possible degradable strains: *Saccharomycopsis lipolytica* GDMCC 2.197, *Yarrowia lipolytica* GDMCC 2.187, *Candida parapsilosis* GDMCC 2.190, *Pichia pastoris* GS115, and *Candida parapsilosis* ACCC 20221. We picked yeast single colonies on YPD plates, inoculated them in 5 mL of YPD liquid medium at 28 °C and 180 r/min for 24 h, and transferred them to 50 mL of fermentation medium for 48 h. The thalli were collected by centrifugation and washed twice in physiological saline; after discarding the supernatant, the samples were vacuum freeze-dried at −45 °C for 48 h to obtain whole-cell catalysts and stored at 4 °C for future use.

Five potential candidates were assayed for the reduction activity of the transformation of 3-keto DON to 3-epi-DON in 500 μL of a solution composed of 20 μg/mL 3-keto-DON and 50 mM phosphate buffer (pH 7.4). The reaction was carried out at 37 °C for 48 h, and terminated by adding an equal volume of methanol; the samples were then analyzed using HPLC, as described above.

The 3-keto-DON degradation rate was calculated using the following formula: Percentage decrease in 3-keto-DON = [1 − (remaining 3-keto-DON concentration/initial 3-keto-DON concentration)] × 100.

### 5.6. Liquid Chromatography–Mass Spectroscopy (LC–MS)

The 3-keto-DON was degraded by purified QDDH for 0 and 48 h incubation at 37 °C, and the products were filtered through a 0.45 μm microporous filter membrane and analyzed by LC–MS. The LC–MS system consisted of an Agilent 1100 LC system (Agilent SB-C18 RRHD, 2.1 × 50 mm, 1.8 µm, Santa Clara, CA, USA) and an Esquire HCT PLUS Ion Trap Mass Detector (Bruker Co., Karlsruhe, Germany). The eluents were composed of acetonitrile and water (16:84, *v*:*v*) and the source conditions were set as follows: ion polarity, positive; nebulizer, 2.0 bar; capillary, 3500 V; charging voltage, 2000 V; end plate offset, −500 V; dry heater, 180 °C; dry gas, 6.0 L/min; scan begin, 20 *m*/*z*; and scan end, 500 *m*/*z*.

### 5.7. Catalytic Epimerization of Deoxynivalenol

Referring to the reaction system described in Section 3 (Analysis of recombinant protein for DON oxidation Activity), the enzyme QDDH was inactivated by incubation at 80 °C for 20 min after reacting at 37 °C for 12 h. Subsequently, 0.1 g/mL *Candida parapsilosis* ACCC 20221 whole-cell catalyst was added to the reaction system. The 3-keto-DON, the product of the first oxidation reaction, was used as the catalytic substrate. The reaction was continued for 6, 12, 24, or 36 h, and was terminated by adding an equal volume of methanol. The DON and 3-keto-DON contents at each time point were analyzed and compared using HPLC.

## Figures and Tables

**Table 2:** 3-keto-DON conversion rate of yeast.

Strains	Conversion Rate/%	Products		Carbonyl Reductase Enzyme Activity in the Literature
		3-epi-DON	DON	
*Yarrowia lipolytica* GDMCC 2.187	99.63 ± 0.05	42.98 ± 0.30	56.65 ± 0.23	96 U/mg, 85 U/mg [53]
*Candida parapsilosis* ACCC 20221	86.59 ± 0.10	51.25 ± 0.76	35.34 ± 0.79	1.27 U/mg [46,47]
*Pichia pastoris* GS115	58.60 ± 3.88	34.94 ± 2.20	23.66 ± 1.69	224.4 U/mg [55]; 27 U/mg [56]
*Saccharomycopsis lipolytica* GDMCC 2.197	37.13 ± 2.67	/	37.13 ± 2.67	96 U/mg, 85 U/mg [53]
*Candida parapsilosis* GDMCC 2.190	30.21 ± 2.63	/	30.21 ± 2.63	1.27 U/mg [46,47];
*Rhodococcus opacus* PD 630	/	/	/	110 U/mg [60]

## Data Availability

All data generated or analyzed during this study are included in this published article.
